# Physiological Chemistry

**Published:** 1898-12

**Authors:** John A. Miller


### Progress in Medical Science.

### PHYSIOLOGICAL CHEMISTRY.

By JOHN A. MILLER, Ph. D.

### INFLUENCE OF ALCOHOL ON DIGESTION.

RUSSEL H. CHITTENDEN, Lafayette B. Wendel and Holmes C. Jackson (*Amer. Jour. Physiol.*, 1898 ; *Jour. Chem. Soc.*, 1898,) say that on the introduction of alcoholic beverages into the mouth of dogs and men, the flow of saliva is transitorily increased and on its introduction into the stomach, the gastric secretion increased in quantity, acidity and proteolytic activity. Alcoholic drinks retard the activity of digestive fluids, but in the living animal this is counterbalanced by the increased secretion of the juices mentioned, as well as by the rapid absorption of the alcohol given ; experiments with test-meals, with and without alcohol, gave practically the same results. There is little or no direct influence on pancreatic or intestinal juices.

---

### INFLUENCE OF BORAX AND BORIC ACID ON NUTRITION.

Russel H. Chittenden and William J. Gies (*Amer. Jour. Physiol.*, 1898 ; *Jour. Chem. Soc.*, 1898,) say that doses of borax up to five grams, even when continued for some time, have no effect on the nutritional changes of the body. Larger doses (5 to 10 grams daily) increase proteid metabolism and larger amounts of nitrogen, sulphuric acid and phosphoric acid are excreted in the urine.

Boric acid up to three grams daily has no effect. In larger doses, borax retards the assimilation of proteid and fatty food, whilst with very large doses there is a diarrheic tendency. Borax decreases the volume of the urine and passing as such into this excretion, raises its specific gravity and renders it alkaline. Both borax and boric acid are rapidly eliminated, and so no cumulative action can result from their daily use. They have no influence on putrefaction in the intestines.

---

### TOXIC ACTION OF ACETYLENE.

U. Mosso and F. Ottolenghi (*Real. Accad. Linc.*, 1898; *Jour. Chem. Soc.*, 1898,) report that experiments were made on dogs, rabbits, rats, sparrows, frogs, tritons and newts. The authors conclude that acetylene present in air in small proportion gives rise, in animals, to a proteid of excitement followed by one of paralysis, during which cardiac and respiratory failure are observed, paralytic symptoms then supervene and death occurs without convulsions.

---

### RHINOLITH OR NASAL CALCULUS.

Poole (*New York Med. Jour.*) reports an interesting case in the person of a young woman, aged 24 years, who consulted him for nasal catarrh. He found hypertrophy of the turbinates and suggested an operation for its relief. Five days afterward he removed a portion of the tampon under an anesthetic, but finally succeeded and cleansed the cavity with hydrozone. Next day, after again cleansing the nostril, he discovered a body which under cocaine he removed with a dressing forceps. It proved to be a calculus. He then thoroughly cleansed and disinfected the cavity with the hydrozone solution, which removed the odor and rendered the cavity wholesome.

The next day two smaller pieces were removed while cleansing and treating the nose. They were loose and seemed as though they had just scaled off from the bed where the larger piece had lain.

The spraying of the nasal cavity with hydrozone, followed by the use of glycozone, constituted the treatment for the next four days, by which time the offensive odor had entirely disappeared, and the parts had assumed a healthy condition.

This concretion formed on the outer side of the inferior meatus, and as it grew larger it obstructed the flow of tears through the naso-lachrymal canal, as evidenced by an overflow of tears from the left eye, which condition ceased immediately after removal of the rhinolith.

